# Dual roles of immunosenescence in cancer immunotherapy

**DOI:** 10.3389/fimmu.2026.1779141

**Published:** 2026-03-06

**Authors:** Ye Yu, Lei Sun, Jiawei Fan

**Affiliations:** 1 West China School of Public Health and West China Fourth Hospital, Sichuan University, Chengdu, Sichuan, China; 2 Department of Radiation Oncology, Sichuan Clinical Research Center for Cancer, Sichuan Cancer Hospital & Institute, Sichuan Cancer Center, Affiliated Cancer Hospital of University of Electronic Science and Technology of China, Chengdu, Sichuan, China; 3 Department of General Specialized, Sichuan Clinical Research Center for Cancer, Sichuan Cancer Hospital& Institute, Sichuan Cancer Center, University of Electronic Science and Technology of China, Chengdu, Sichuan, China

**Keywords:** cancer immunotherapy, immune cell, immunosenescence, senescence, TME (tumor microenvironment)

## Abstract

Cellular senescence of tumor-infiltrating immune cells represents a hallmark of tumorigenesis, characterized by functional decline and severely compromised anti-tumor immunity. A growing body of evidence indicates that immunosenescence is not only a significant biological phenomenon within the tumor microenvironment (TME) but also serves as a therapeutic target with dual regulatory potential. Previous reviews have thoroughly examined the biomarkers associated with immunosenescence and its overarching impact on aging and disease. Nonetheless, comprehensive analyses of its specific and dual roles in cancer immunotherapy outcomes are still lacking. Consequently, a novel paradigm has been proposed for targeting immunosenescence: modulating the function of senescent immune cells may offer innovative cancer treatment strategies. This review synthesizes the bidirectional relationship between immune cell senescence and tumor progression, systematically examining the interactions between senescent immune cells and tumor advancement, and underscores the key molecular mechanisms driving immune senescence within the TME. By elucidating the characteristics and functions of immune cell senescence in tumor development and immune evasion, we aim to uncover new therapeutic perspectives and highlight potential targets for cancer immunotherapy.

## 1 Introduction

In recent years, tumor immunotherapy has revolutionized cancer treatment. Unlike conventional approaches such as chemotherapy and radiotherapy, immunotherapy leverages the host’s immune system to eliminate tumor cells. Notably, therapies targeting immune checkpoints—particularly anti-CTLA-4 and anti-PD-1/PD-L1 monoclonal antibodies—have achieved breakthrough progress in oncology (1). Among these, anti-PD-1/PD-L1 monotherapies (e.g., pembrolizumab, nivolumab, atezolizumab) have shown significant efficacy against various cancers, including lung, breast, and colorectal malignancies (2–5). Critically, chimeric antigen receptor T-cell (CAR-T) therapy uses genetic engineering to modify T cells, yielding remarkable results in treating hematological cancers such as acute lymphoblastic leukemia and non-Hodgkin lymphoma (6, 7). Beyond these, oncolytic virus therapy and tumor-infiltrating lymphocyte (TIL) therapy have also demonstrated promising potential in clinical studies, further expanding the arsenal of immunotherapeutic options (8).

## 2 The development of immune cell senescence

Immunosenescence refers to the process where immune cells progressively lose proliferative capacity and enter a static state under chronic stimulation (9–11). Unlike the aging of somatic cells, immunosenescence is a complex process characterized by unique mechanisms, including age−related thymic involution and T−cell exhaustion driven by prolonged antigen exposure. Studies indicate that thymic involution is a significant contributor to immune senescence. With advancing age, thymic function progressively declines, resulting in reduced T−cell output and consequently impairing overall immune competence (12). Furthermore, chronic antigen exposure drives T cells—particularly memory T−cell subsets—into an exhausted state accompanied by replicative senescence (13).

Notably, aging itself triggers a chronic, low−grade inflammatory state termed “inflammaging,” which in turn can induce senescence in diverse immune cells such as T cells, B cells, and NK cells, forming a vicious cycle that accelerates immune decline (14, 15). Senescent cells secrete abundant inflammatory factors (e.g., IL-6, TNF-α), further driving chronic inflammation and tumorigenesis (9). Critically, these cells in the tumor microenvironment lose tumor-targeting functionality while secreting SASP factors that promote tumor proliferation, migration, and invasion.

Intriguingly, recent findings reveal senescent T cells can acquire NK cell-like cytotoxicity against tumors, highlighting the dual regulatory role of immunosenescence (16). Given this complexity, dissecting its molecular networks will enable: combined strategies to reverse immune senescence, and novel senolysis-based therapeutic targets. Thus, systematically elucidating the biological significance of immunosenescence in tumor development is crucial for advancing cancer therapy.

## 3 Characteristics and mechanisms of immune cell senescence

### 3.1 Molecular markers of immune cell senescence

P16 and P21 are established cellular senescence markers that function by inhibiting cyclin-dependent kinases (CDKs). These proteins also regulate the senescence-associated secretory phenotype (SASP) in aging immune cells. Elevated p16/p21 expression triggers secretion of inflammatory cytokines and chemokines (e.g., IL-6, TNF-α), establishing paracrine signaling that alters the tissue microenvironment and accelerates aging-related pathologies. Studies have demonstrated cell-type-specific impacts: in T cells, p16 upregulation correlates with proliferative arrest and senescence acquisition. And in B and NK cells, p16/p21 activation induces cell cycle blockade, senescent state transition, and compromised cytotoxic activity (17). Furthermore, studies have indicated that CD57 can serve as a key marker for the terminal differentiation and replicative senescence of human T cells and NK cells (18, 19). KLRG1 is another important marker associated with immune aging, and its expression typically signifies that T cells have entered the terminal differentiation stage, accompanied by cell cycle arrest and loss of clonal expansion ability (20).

### 3.2 Induction mechanisms of senescent immune phenotypes

Accumulation of irreparable DNA damage constitutes the core mechanism driving cellular senescence. Under physiological conditions, the DNA damage response (DDR) system safeguards genomic stability by activating cell cycle checkpoints and initiating repair programs through rigorous regulation of the p53-p21CIP signaling pathway (21–23). However, under pathological conditions (e.g., sustained exposure to genotoxic stressors), persistent DNA damage overwhelms DDR functionality, ultimately triggering cellular senescence.

Excessive reactive oxygen species (ROS) constitute a major inducer of DNA damage and cellular senescence. While cells normally maintain redox homeostasis through tightly regulated antioxidant systems, aging disrupts this balance, causing ROS accumulation that impairs cellular function (24, 25). Notably, ROS-mediated guanine oxidation generates 8-oxoguanine (8-oxoG)—a predominant DNA lesion that accumulates in aging tissues (26).

In addition, studies in immunosenescence further demonstrate that ROS levels strongly correlate with aging in human naïve and central memory CD8^+^ T cells. Remarkably, ROS scavenging inhibits telomere shortening in these cells, delaying senescence (27). Moreover, DNA damage induces epigenetic reprogramming. Age-associated accumulation of γH2AX foci—definitive markers of DNA double-strand breaks—occurs in both hematopoietic stem/progenitor cells and T lymphocytes, suggesting conserved DNA damage responses during aging (28).

Endoplasmic reticulum (ER) stress and the unfolded protein response (UPR) significantly contribute to immune cell aging. The UPR—activated primarily through three major sensors (IRE1α, PERK and ATF6α)—serves as a critical cellular pathway for resolving ER stress and maintaining proteostasis (29). The inflammatory microenvironment constitutes a pivotal component of the tumor microenvironment. While moderate inflammation can activate host immunity and suppress tumor growth, excessive inflammatory responses may promote tumorigenesis and metastasis. Critically, chronic inflammation drives functional decline and senescence in immune cells through proinflammatory cytokine release, which: activates senescence pathways, induces cell cycle arrest and causes cellular dysfunction (30). Under chronic inflammation, immune cells exhibit metabolic reprogramming and elevated oxidative stress, which collectively accelerate immunosenescence (14). Notably, DNA-damaging chemotherapy can prematurely induce senescence. Studies have shown that chemotherapy can accelerate the aging of Vδ2pos γδ T cells in patients with colorectal cancer liver metastases, leading to the accumulation of CD57-positive terminally differentiated cells and impairing their anti-tumor function (31).

## 4 Role of immune cell senescence in tumor development

In the tumor microenvironment, the functional decline of T cells—particularly exhaustion and senescence—constitutes a key factor limiting the efficacy of immunotherapy (11). These two tumor−induced dysfunctional states often coexist in patients, directly undermining treatment responses by impairing antitumor immunity and sustaining an immunosuppressive milieu. Although both exhausted and senescent T cells exhibit compromised effector functions, they differ fundamentally in their origins, developmental paths, and molecular regulation. At present, the definitions of “senescence” and “exhaustion” remain prone to confusion due to partially overlapping features; however, they are governed by distinct regulatory networks that dictate their differentiation trajectories and modes of functional impairment. It is noteworthy that T cell exhaustion was originally defined in models of chronic viral infection, characterized by persistent high expression of inhibitory receptors such as PD−1, CTLA−4, Tim−3, and LAG−3. In contrast, cellular senescence represents a broader biological program typically triggered by intrinsic stresses like replicative aging, DNA damage, or oncogenic stress, and is marked by irreversible cell−cycle arrest, resistance to apoptosis, and an active senescence−associated secretory phenotype (SASP) (11, 32) (**Table 1**).

**Table 1:** Characteristics comparison between exhausted and senescent T-cells.

Characteristics	Exhaustion	Senescence
Cause	Persistent exposure to antigens	Repetitive stimulation; DNA damage agents; stress signals
Typical feature	Proliferative activity ↓ p27, p15 ↑; cyclin E-Cdk2, Cdc25A ↓	Proliferative activity ↓ p16, p21, p53 ↑; DNA damage-associated molecules ↑; Telomere length, telomerase activity ↓; SA-β-gal activity ↑
Surface marker	PD-1, CTLA-4, Tim-3, LAG-3, BTLA, TIGIT, CD244, CD160, CD39, 4-1BB ↑	CD27, CD28 ↓; CD57, KLRG1, Tim-3, TIGIT, CD45RA, lncRNA NEAT1, MALAT1 ↑
TCR signaling machinery	Lck, ZAP70 ↓	Lck, ZAP70, DLG1, Lat, SLP-76 ↓
Cytokine profile	Early stage: IL-2 ↓; Intermediate stage: TNF ↓ Terminal stage: IFN-γ, β-chemokines↓	SASP, IL-6, IL-8, IFN-γ, TNF ↑; IL-10, TGF-β ↑;
Transcriptional profile	NFAT, Nr4a, Blimp-1, BATF, FoxP3 ↑; Progenitor subset: T-bet^high^Eomes^low^PD-1^int^; Terminal subset: T-bet^low^Eomes^high^PD-1^high^	FoxP3 ↑
Epigenetic change	Exhaustion-associated DNA methylation programs	SAHF ↑
Metabolic alternation	Glycolysis ↓; Mitochondrial biogenesis ↓; Reactive oxygen species ↑	Glycolysis ↑; Mitochondrial biogenesis ↓; Reactive oxygen species ↑
Functional alteration	Cytotoxic activity ↓; Effector molecule: GzmB ↓	Cytotoxic activity ↓; Suppressive functions ↑ Effector molecules: perforin, GzmB ↓

Research reveals that senescent immune cells exhibit dual functionality in tumor progression: (1) tumor-promoting effects: driving inflammation and suppressing immune responses (33–35), (2) protective roles: potentiating anti-tumor immunity (16, 36). Consequently, characterizing the context-dependent functions of cellular senescence—across cancer types and disease stages—is essential for developing targeted therapeutic strategies (**Table 2**).

**Table 2:** Role of senescent T Cells in tumor progression.

Characteristics	Promoting tumor progression	Inhibiting tumor progression
Phenotypic Features	Senescent phenotype: proliferation↓, telomere attrition↑; senescence markers: (CD57, KLRG1) ↑, TCR signaling (Lck/LAT) ↓.	Acquisition of innate NK-like functions: NK receptors (NKG2D, NKG2A)↑
Direct Functions	SASP secretion↑; co-stimulators (CD27/CD28/CCR7) ↓; inhibitory markers (KLRG1/TIM3) ↑; effector molecules (perforin/granzymes) ↓	Antigen-independent cytotoxicity↑; cytotoxic granules ↑
Mechanisms	Inflammatory cytokines (TNF-α, IL-1β, IL-6) create immunosuppressive TME; IL-10/TGF-β suppress effector cells; Treg/MDSC expansion	Enhanced anti-tumor immunity in aged hosts (e.g., lung metastasis in aged mouse models↓).
References	(33–35)	(16, 36)

### 4.1 Tumor-induced immunosenescence

Studies prove that tumors employ diverse strategies to dysregulate T-cell glucose and lipid metabolism, thereby suppressing anti-tumor immunity (37–41). Notably, tumor-derived Tregs enhance immunosuppression through: (1) competitive glucose consumption against effector T cells, (2) hyperactivated glycolysis, (3) induction of senescence in dendritic and T cells in the tumor microenvironment (42–44). Moreover, tumor cells themselves exhibit increased glucose/glutamine uptake, causing nutrient depletion and metabolite accumulation (45–47). For example, melanoma models of adoptive immunotherapy demonstrate that both tumor cells and Tregs induce senescence in transferred tumor-specific T cells, compromising therapeutic efficacy (48).

**Figure 1:**
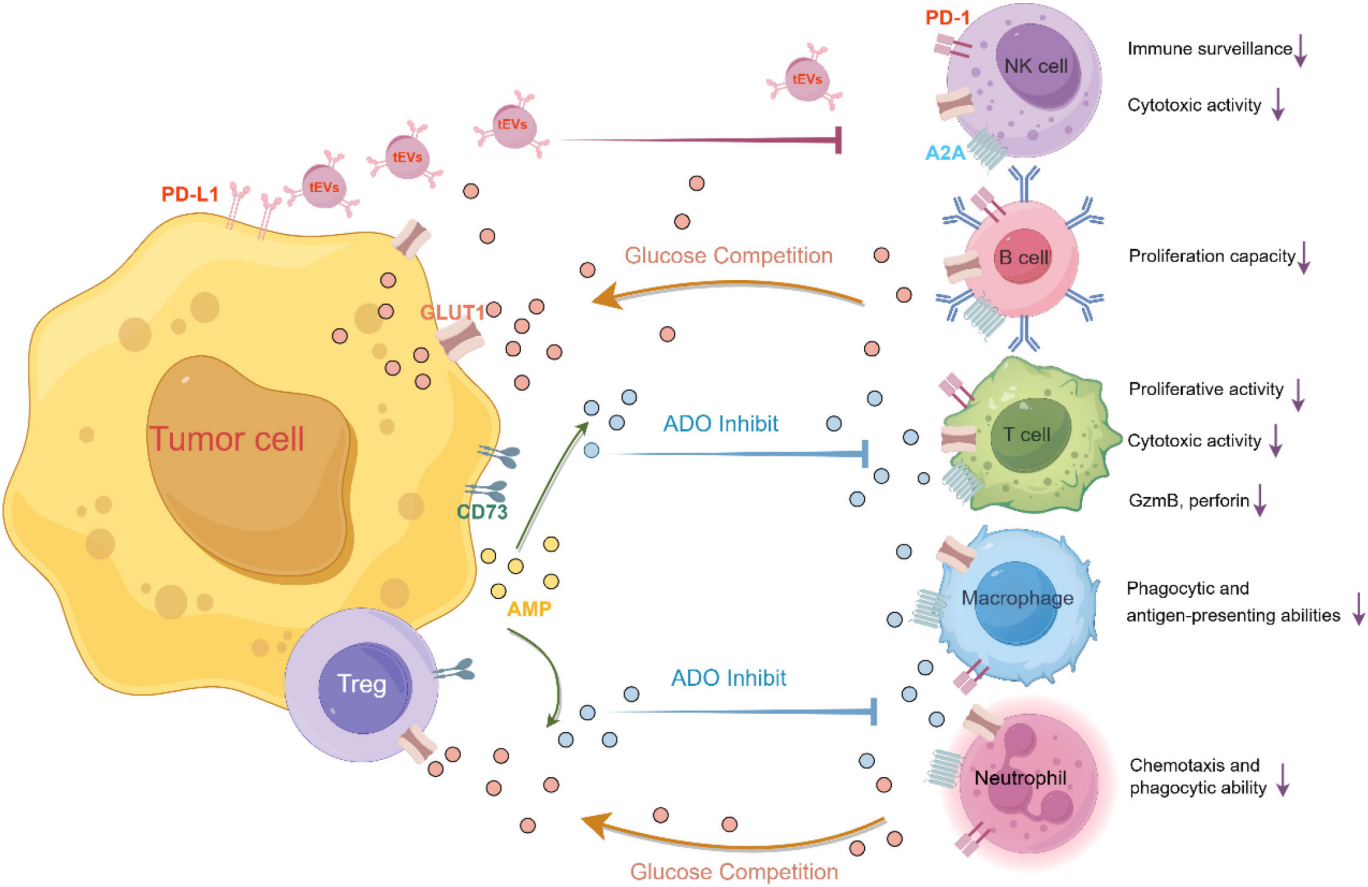
Tumors promote immune cell senescence via multiple pathways. (1) PD-L1-mediated metabolic reprogramming: tumor-derived extracellular vesicles (tEVs) deliver PD-L1, activating the CREB/STAT pathway to reprogram effector T cell lipid metabolism. This induces systemic T cell senescence and immunosuppression. (2) Glucose competition: regulatory T cells (Tregs) and tumor cells competitively consume glucose, restricting effector T cell access and impairing function. (3) Metabolite-induced senescence: Tregs use tumor-derived metabolites (e.g., AMP, adenosine (ADO)) to directly induce senescence in effector T cells, facilitating tumor immune evasion.

Additionally, studies indicate that regulatory T cells (Tregs) and various tumor cells—including those from breast cancer, melanoma, colon cancer, prostate cancer, ovarian cancer, and head and neck cancer—can induce T cell senescence via tumor-derived metabolites (e.g., cyclic adenosine monophosphate [cAMP], IDO, adenosine, and lactate) to evade immune surveillance (48–50). These metabolites promote senescence through multiple mechanisms: (1) cAMP directly suppresses tumor-specific T cell function and triggers senescence via ATM-related DNA damage response (48, 51), (2) adenosine elevates intracellular cAMP through receptor binding, accelerating CD8^+^ T cell senescence while reducing proliferative capacity, telomerase activity, and CD28 expression (46, 52). Notably, tumor-derived exosomes (tEVs) carrying RNA, DNA, lipids, proteins, and metabolites critically regulate tumor/immune cells and sustain a hypoxic, immunosuppressive microenvironment (53–55). For example, (1) tEV-delivered PD-L1 induces T cell lipid metabolism reprogramming by activating the CREB/STAT axis, systemically driving senescence and immunosuppression (55); (2) The transfer of CD57 from glioblastoma stem cells to CAR-T cells induces senescence in the latter (56); (3) CD133-specific CAR-T cells eliminate tumor stem cells but concurrently upregulate CD57, ultimately senescing therapeutic T cells (57). Collectively, these processes impair T cell anti-tumor function and facilitate immune escape (**Figure 1**).

### 4.2 Senescent T-cell: phenotypic signatures and molecular drivers

T cell senescence represents a key aspect of immune system aging. With advancing age, systemic alterations occur in T cell composition and function, characterized by: (1) declining naive T cell populations; (2) expanded terminally differentiated CD27^−^CD28^−^ memory T cells; (3) imbalanced naive-to-memory T cell ratio. Moreover, these CD27^−^CD28^−^ memory T cells exhibit senescence-like features, including proliferation deficiency, telomere attrition, suppressed telomerase activity and upregulation of senescence markers (CD57, KLRG1, γH2AX) (58). Concomitantly, key TCR signaling molecules (Lck/LAT) are downregulated, while NK receptors (NKG2D, NKG2A, CD16) show dysregulated upregulation (16, 59) (**Table 2**).

However, senescent T cells do not merely represent defective terminal populations. Paradoxically, they exhibit dual functions in tumor progression (60): (1) tumor-suppressive role: senescent immune cells can inhibit tumor development by enhancing tumor cell clearance; (2) mechanistic evidence: aged murine CD8^+^ T cells display senescence-like phenotypes with elevated SA-β-gal activity, antigen-independent cytotoxic activity resembling NK cells, and upregulated cytotoxic granules and NK receptors post *in vitro* activation. In metastatic B16 melanoma models, aged mice (≥30-week-old) showed significantly suppressed lung metastasis compared to young controls (6–10-week-old) (16, 61). Which indicates that aged effector CD8^+^ T cells may acquire innate NK-like functions (e.g., antigen-independent cytotoxicity) to promote antitumor immunity in aging hosts.

Also, the senescence-associated secretory phenotype (SASP) from senescent immune cells fuels tumor progression through chronic inflammation and immunosuppression. Key mechanisms are included as follows,

1. Direct immune suppression (62): downregulating immune cell co-stimulatory receptors: CD27, CD28, and CCR7; upregulating inhibitory markers KLRG1, CD57 and TIM3; reducing effector molecules perforin and granzymes.
2. Cytokine-mediated amplification (63): upregulated inflammatory cytokines (TNF-α, IL-1β, IL-6) from senescent immune cells establish immunosuppressive tumor microenvironments (TME), facilitating tumor proliferation.
3. Paracrine cell dysfunction (64): enhanced secretion of IL-10/TGF-β inhibitory cytokines directly impairs Th1, Th17, CD8+T cells and dendritic cells, thereby amplifying tumor immunosuppression.
4. Immunosuppressive cell expansion (64): SASP mediators drive the infiltration and expansion of Tregs and MDSCs within the TME, and enhance their suppressive potential, thereby favoring a vicious cycle of immunosuppression.
5. Intrinsic T cell dysregulation with aging:even without stimulation, aged naive CD8^+^/CD4^+^ T cells exhibit: loss of stemness accompanied with reduced expression of stemness-maintaining genes (FoxO1, CCR7, IL-7R, Lef1, Tcf7, Bach2), and premature differentiation with impaired function via aberrant activation/differentiation pathway (63) (**Table 3**).

**Table 3:** Comparisons of aged T cells, B cells, NK cells, macrophage and neutrophil in metabolic and epigenetic characteristics.

Cell type	Surface markers	Cytokine profile	Metabolic alterations	Functional alterations	Ref
T cell	p16, p21, CD57, KLRG-1, TIM-3, TIGIT, CD45RA, CTLA-4, NKG2D, NKG2A, CD16 ↑ CD27, CD28, CCR7 ↓	IL-6, IL-8, IFN-γ, TNF-α, IL-10, TGF-β ↑ IL-2, IL-7 ↓	Glycolysis ↑ Mitochondrial biogenesis ↓ ROS ↑	Proliferative activity ↓ Cytotoxic activity ↓ Suppressive functions ↑	(12, 16, 35, 50, 65–70)
B cell	CD11c, CD11b ↑ CD21, CD35, CD23, CD43, CD93 ↓	IL-6, TNF-α ↑	ROS ↑ Glycolysis ↑	Proliferation capacity ↓ pro‐inflammatory cytokine ↑	(71, 72)
NK cell	CD56^dim^/CD16^+^ ↑ CD56^bright^/CD16^+^ ↓	PD-1↑ IFN-γ, GZMB ↓	mTOR ↑ ROS ↑	Cytotoxic activity ↓ Immune surveillance function ↓	(63, 64, 73–75)
Macrophage	CD38, IBA-1, CD40, COX2, RACK1, ABC1, p16, p21, Arg1, CD206, CXCR1, CD38 ↑ TLR, MHC II, CD86 ↓	BMP2, CCL2, CCL7, CCL8, CCL24, IL10 CXCL13 ↑	OXPHOS ↓ Arginine metabolism ↑	Phagocytic and antigen-presenting abilities ↓	(76–82)
Neutrophil	CD11a/CD11b ns, CXCR2 ↓	IL-8, MMP-9 ↑, NETs ↓	Glycolysis ↑ ROS ↑	Chemotaxis and phagocytic ability ↓ N2-tumor-associated neutrophils ↑	(83–85)

### 4.3 Senescent B-cell

B cells are integral to the maintenance of humoral immune responses within an effective immune system. They achieve this not only through the production of high-affinity antibodies, which are crucial for vaccination strategies, but also by supporting the functions of other immune system components. In the context of aging, B cells assume a multifaceted role; they contribute to immune responses by secreting proinflammatory cytokines, yet their functional decline can lead to immune escape and tumorigenesis (35). Research indicates that with advancing age, the number of mature B cells in human peripheral blood diminishes, while the proportion of naive B cells and IgM memory B cells remains unchanged, resulting in an overall reduction in the total number of B cells (73). Furthermore, in elderly individuals, there is a marked increase in SA-β-gal activity and the senescence-associated secretory phenotype (SASP) in B cells, accompanied by elevated secretion of IL-6 and TNF-α. This is coupled with a significant reduction in antigen sensitivity to foreign antigens and a diminished capacity to produce antigen-specific antibodies, thereby impairing the body’s response to vaccines and resistance to infections. These changes are among the common indicators of immune senescence in the elderly population (74) (**Table 3**).

Given that tertiary lymphoid structures are key sites where B cells and T cells are locally activated and generate effective anti-tumor immune responses, age-related B cells may be an important mechanism affecting responses to immunotherapy (75). In hepatocellular carcinoma models, T-bet-expressing age-associated B cells (ABCs) actively promote tertiary lymphoid structures (TLS) expansion, whereas inhibiting ABC differentiation reduces TLS formation and suppresses tumor progression (86). Mechanistically, aged CD4^+^ T cells drive ABC accumulation and TLS expansion through the CD153/CD30 signaling pathway and cytokine secretion (IL-21, IFN-γ), suggesting that T cell-B cell interactions are critical for maintaining TLSs during aging (87).

### 4.4 Senescent NK-cell

Natural killer (NK) cells play a critical role in immune surveillance against senescent cells and are among the immune system’s early responders. However, with advancing age, key NK cell functions—including antigen presentation, endocytosis, and interferon (IFN) production—decline. This dysfunction is associated with increased susceptibility to infections, malignancies, inflammation, and the accumulation of senescent cells. NK cells are broadly categorized into two phenotypically and functionally distinct subpopulations based on CD56 expression: CD56^dim^ and CD56^bright^. The CD56^dim^ subset represents mature NK cells characterized by strong cytotoxic activity. In contrast, the CD56^bright^ subset, considered less mature, exhibits limited cytotoxicity but can be activated by interleukin-2 (IL-2) to proliferate (76). Age-related changes significantly impact these subsets. Specifically, the ability of CD56^bright^ NK cells in the elderly to respond to IL-2 is impaired, inhibiting their proliferation. Concurrently, the CD56^dim^ subset expands, resulting in a significant redistribution of NK cell subpopulations (76, 88). Notably, while the overall cytotoxic capacity of circulating NK cells often remains unchanged in aging due to the increased number of mature (CD56^dim^) cells, there is an age-related impairment in the per-cell cytotoxic function, attributed to reduced expression of activating receptors (89) (**Table 3**).

Interestingly, studies in aging mouse models reveal an opposite trend concerning NK cell composition compared to humans. In most peripheral tissues of aged mice, both the mature NK cell subset (characterized as CD11b^+^ CD27^−^) and total NK cell content are significantly reduced. However, this reduction is not observed in the bone marrow. Analysis of NK cell developmental stages within the bone marrow showed similar numbers of early developmental stage NK cells in aged mice compared to young, but a decrease in cells at the terminal maturation stage. This finding indicates impaired terminal maturation in aged mice (90). Consequently, the reduction in circulating mature NK cells may stem from both low maturation efficiency within the bone marrow and reduced egress of mature NK cells from this compartment.

The reasons for these striking differences in NK cell aging trajectories between mice and humans remain poorly understood. Angela R. Manser and colleagues proposed that factors such as species lifespan disparities, inherent differences in NK cell biology, the pathogen-free laboratory environment (potentially leading to a lack of immune memory formation), and the human reliance on peripheral blood analyses (ignoring potential tissue-specific variations) might contribute to these contrasting observations (91).

### 4.5 Senescent macrophages

#### 4.5.1 Impaired metabolic function and immunosuppressive phenotype

Due to significant downregulation of glycolysis and mitochondrial oxidative phosphorylation (OXPHOS), aged macrophages enter an energy-depleted state. This metabolic impairment inhibits their phagocytic and antigen-presenting capacities (92). Consequently, these cells often exhibit immunosuppressive properties within the tumor microenvironment (TME), promoting tumor progression (77, 78) (**Table 3**).

#### 4.5.2 Altered polarization and distribution

Substantial evidence indicates that immunosuppressive M2-like tumor-associated macrophages (TAMs) are highly enriched in the spleen, bone marrow, and lymphoid tissues of aged mice, where they facilitate tumor progression in the aging milieu (79, 93, 94). However, studies reveal that the phenotype of aged macrophages in the TME does not conform to classical M1 (characterized by high iNOS expression) or M2 (characterized by high Arg1 expression) polarization states (80) (**Table 3**).

#### 4.5.3 Declining recognition and presentation capabilities

Aging is associated with reduced expression of Toll-like receptors (TLRs) on macrophages (81), alongside decreased levels of antigen-presenting machinery components, including MHC class II molecules and the co-stimulatory receptor CD86. This collectively impairs their ability to effectively present antigens (**Table 3**).

#### 4.5.4 Enhanced inflammatory output

Notably, several studies demonstrate that aged macrophages exhibit increased production of inflammatory cytokines (82, 95).

Identifying specific markers capable of selectively distinguishing aged or senescent macrophages is crucial for developing targeted therapies. For example, (1) classic Senescence markers: beyond established biomarkers of cellular senescence such as p16 INK4a and senescence-associated β-galactosidase (SA-β-Gal); (2) emerging marker: CD38 is highly expressed in senescent-like macrophages and serves as a valuable biomarker for this population (83, 84, 96); (3) aging-associated upregulation: furthermore, genes and proteins associated with aged macrophages, including Ionized calcium-binding adapter molecule 1 (IBA-1, a macrophage activation marker), CD40, cyclooxygenase-2 (COX2), are significantly upregulated with aging (97).

### 4.6 Senescent Neutrophils

Neutrophils undergo immunosenescence within the chronic, low-grade inflammatory microenvironment. Their metabolic dysfunction and impaired immune activity collectively underlie the promotion of tumor development (30, 98).

#### 4.6.1 Key features of aged neutrophils

Studies proved that the key features of aged neutrophils are as following: (1) preserved quantity but altered quality: physiological aging exerts no significant impact on circulating neutrophil numbers or the expression of major surface markers (e.g., CD11b, CD62L) (98, 99). However, it induces multiple functional impairments: such as reduced phagocytic ability (100), impaired chemotactic migration (101), increased apoptosis (85), and dysregulated Toll-like receptor (TLR) signaling (102) (**Table 3**).

#### 4.6.2 Mechanisms driving inflammation and dysfunction

In aging mice, CXCL1 disrupts neutrophil migratory patterns, contributing to tissue damage, distal organ inflammation, and liver neutrophil infiltration. This promotes ROS-mediated, age-related inflammation. Concomitantly, age-associated remodeling of the bone marrow microenvironment exacerbates neutrophil dysfunction (103). These factors collectively fuel chronic inflammation in the elderly. And studies reveal significantly higher infiltration levels of pro-tumoral N2 tumor-associated neutrophils (TANs) within the TME of aged mice compared to young mice, which markedly accelerates tumor growth (104, 105) (**Table 3**).

### 4.7 Senescent dendritic cells

DCs are important antigen-presenting cells that play a key role in the production and maintenance of tolerance of immunity. DCs comprise of two major subsets, myeloid DCs, also known as conventional DCs (cDCs); and plasmacytoid DCs (pDCs) that are of a lymphoid lineage (106, 107). It has been found that the function of DCs is impaired with age, specifically by a significant decrease in antigen presentation function due to mitochondrial dysfunction within the cell and weak chemotaxis during migration to lymph nodes, which may be due to its weakened response to chemokines (107, 108). More importantly, when senescent conventional DCs (CDCs) are stimulated by foreign antigens, the secretion of pro-inflammatory cytokines (such as IL-12) is reduced, resulting in a weakened T cell response. The plasmacytoid DCs (PDCs) also significantly reduced the secretion ability of IFN-α in response to viral infection (107). In addition, dendritic cells in the elderly also exhibit dysfunction in interactions with other immune cells. For example, the interaction between aged DCs and natural killer cells (NKs) is weakened, leading to insufficient activation of NKs, which affects the clearance capacity of tumors (109).

## 5 Targeting strategies for T cell senescence

A deepening understanding of immune cell senescence within the tumor microenvironment (TME) has spurred the development of therapeutic strategies targeting this phenomenon, positioning them as a crucial frontier in tumor immunotherapy research. Critically, immune cell senescence not only compromises immune cell function but may also actively promote tumorigenesis and progression. Therefore, developing therapies that specifically counteract immune cell senescence holds significant potential for improving tumor treatment efficacy.

Inducing T cell senescence represents a key mechanism of immune escape in malignancies. Consequently, designing interventions that target senescence regulatory pathways to precisely control the differentiation state and effector function of tumor-specific T cells has emerged as a central objective for enhancing anti-tumor immunity.

The targeted removal of senescent cells represents a promising therapeutic approach for tumors. Eliminating these cells restores immune homeostasis within the tumor microenvironment (TME), thereby enhancing immunotherapy efficacy. The key strategies are as follows(**Table 4**):

**Table 4:** Summary of therapeutic strategies targeting immunosenescence.

Strateges	Targets	Function
Dasatinib + Quercetin (D+Q)	SRC kinase, PI3K/AKT signaling; PI3K-dependent regulation of BCL-XL	treating age-related idiopathic pulmonary fibrosis(IPF) (110)
CD153 vaccination	CD153	preventing senescent T cell accumulation in adipose tissue and ameliorating obesity-related metabolic dysfunction in mice (111)
uPAR CAR-T	uPAR	murine lung adenocarcinoma models (112)
TLR8 Agonism	TLR8-MyD88 signaling pathway	disrupts the Treg-mediated induction of effector T cell senescence within tumors (42, 113, 114)
iBFAR2	BFAR	restored the anti-tumor functionality of aged CD8^+^ T cells (115)
rapamycin	mTOR	reduce p16 and p21 expression in CD3^+^ T cells (9)
ILT4 Inhibition	ILT4	reduces T cell senescence and enhances immunotherapy efficacy in breast cancer and melanoma models (116)
T-Lip/ATK cells	cPLA2α	delay senescence (117)
Disrupting tEV signaling	inhibiting tumor EV synthesis or blocking CREB signaling, cholesterol synthesis, and LD formation	prevents tEV-mediated T cell senescence (55)
pharmacological inhibition (JQ-1 or molibresib/I-BET762) or siRNA knockdown of BRD4	downregulates SASP factor expression	alleviates pathological lipid accumulation in aged macrophages (118)
quercetin	suppresses SASP components	inhibiting macrophage senescence (65)

### 5.1 Direct senescent cell elimination strategies

Pharmacological senolytics: (1) Navitoclax reverses myeloid-derived immunosuppression in the TME. *In vitro*, eliminating senescent myeloid cells restores CD8^+^ T cell proliferation; *in vivo*, it overcomes immunotherapy resistance (66). And a phase II trial demonstrated superior efficacy of Navitoclax plus rituximab versus rituximab alone in chronic lymphocytic leukemia patients (67). Navitoclax Pro-drug (Nav-Gal): Conjugation to β-galactosidase (SA-β-gal substrate) enhances its specificity for senescent cells, improving systemic safety (68). (2) Dasatinib + Quercetin (D+Q) pioneering senolytic combination targets pro-survival pathways in senescent cells, Dasatinib inhibits key nodes (e.g., SRC kinase, PI3K/AKT signaling), and Quercetin modulates anti-apoptotic pathways (e.g., PI3K-dependent regulation of BCL-XL). In all, D+Q has demonstrated significant efficacy in treating age-related idiopathic pulmonary fibrosis(IPF) (110).

Immunological Approaches: (1) CD153 vaccination: Induces durable immunity preventing senescent T cell accumulation in adipose tissue and ameliorating obesity-related metabolic dysfunction in mice (111). (2) uPAR-Specific CAR-T Cells: Emerging studies show these engineered T cells effectively eliminate senescent cells *in vitro* and *in vivo*, significantly prolonging survival in murine lung adenocarcinoma models (112).

### 5.2 Targeting senescence induction and reversal in immunotherapy

Beyond direct senolysis, modulating senescence-inducing pathways or reversing senescent phenotypes in immune cells shows promise for cancer immunotherapy.

TLR8 Agonism to Counteract Treg-Mediated Immunosuppression: Toll-like receptor 8 (TLR8) is a key receptor expressed specifically by thymus-derived natural CD4^+^CD25^+^ Tregs and tumor-infiltrating Treg subsets. Activating Tregs via TLR8 ligands robustly triggers the TLR8-MyD88 signaling pathway, and this activation disrupts the Treg-mediated induction of effector T cell senescence within tumors and reverses their immunosuppressive function (42, 113, 114). Concurrently, TLR8 signaling directly targets diverse tumor cell types, inhibiting their capacity to induce T cell senescence (48, 50).

### 5.3 Restoring tissue-resident memory T cell function via BFAR inhibition

Studies revealed that CD8^+^ T cells from aged donors exhibit significantly impaired differentiation into tumor-infiltrating TRM cells compared to young donor cells in Rag1^−+−^ mouse tumor models. And BFAR gene knockout in aged CD8^+^ T cells or treatment with iBFAR2 (a specific small-molecule BFAR inhibitor) effectively restored the anti-tumor functionality of aged CD8^+^ T cells. Crucially, iBFAR2 potentiates the efficacy of anti-PD-1 antibody therapy, demonstrating significant synergistic anti-tumor effects (115).

### 5.4 Modulating mTOR activity to ameliorate immune aging

Rapalogs (e.g., everolimus) inhibit mTOR activity and have been shown to enhance responses to influenza vaccination and reduce infection rates in the elderly (69), suggesting broader immunorestorative effects. And in murine models, mTOR inhibitors like rapamycin reduce p16 and p21 expression in CD3^+^ T cells, indicating their potential to mitigate T cell immunosenescence (9).

### 5.5 Regulating dysregulated lipid metabolic to improve T cell senescence and therapy resistance

Tumor cells disrupt lipid metabolism homeostasis in effector T cells, inducing cellular senescence that undermines adoptive T cell therapy efficacy and facilitates tumor immune escape, which includes the following key molecular mechanisms:

1. Senescent T cell metabolism: compared to normal cells, senescent T cells exhibit dysregulated lipid metabolism upon activating the MAPK ERK1/2 and STAT1/3 pathways through interactions with tumor cells and Tregs (116).
2. ILT4-mediated senescence: immunoglobulin-like transcript 4 (ILT4), expressed by tumor and myeloid cells, activates MAPK ERK1/2 signaling to promote tumor lipid accumulation and drive effector T cell senescence. And critical evidence suggesting that blocking ILT4 significantly reduces T cell senescence and enhances immunotherapy efficacy in breast cancer and melanoma models (116).
3. cPLA2α-dependent pathway: STAT1/3 and MAPK activation upregulates cytosolic phospholipase A2α (cPLA2α), elevating free fatty acid levels and triggering lipid droplet (LD) accumulation, ultimately inducing senescence (70, 71). This dysregulation extends to cholesterol synthesis/transport and fatty acid synthase (FASN)-mediated pathways (72).
4. tEV/PD-L1 signaling: PD-L1-enriched tumor-derived extracellular vesicles (tEVs) activate cAMP response element binding protein (CREB) and STAT signaling, promoting DNA damage and hyperactive lipid metabolism in T cells, driving senescence and immunosuppression (55).

### 5.6 Other therapeutic interventions on immunosenescence

1. ILT4 Inhibition: Restores T cell function and synergizes with immunotherapy (116).
2. cPLA2α targeting: the specific inhibitor arachidonoyl trifluoromethyl ketone (ATK) enhances T cell anti-tumor activity in melanoma/breast cancer models (72). And T-Lip/ATK cells (T cells coated with ATK-loaded liposomes via membrane anchoring) downregulate cPLA2α, clear LDs, rebalance lipid metabolism, delay senescence, and boost anti-tumor efficacy (117).
3. Disrupting tEV signaling: inhibiting tumor EV synthesis or blocking CREB signaling, cholesterol synthesis, and LD formation prevents tEV-mediated T cell senescence. This approach enhances adoptive T cell therapy and anti-PD-L1 immunotherapy in melanoma models (55).

Together, these results suggest that targeting T cell lipid metabolism and senescence could address resistance to cancer immunotherapy.

## 6 Targeting macrophage senescence in cancer immunotherapy

Beyond T cell senescence, therapeutic strategies focusing on senescent macrophages show significant anti-tumor potential. Key findings include:

1. SASP-mediated tumor progression: the senescence-associated secretory phenotype (SASP) remodels macrophage phenotype and function, driving recruitment of tumor-associated macrophages (TAMs) to the tumor microenvironment, synergistic immunosuppression, therapeutic resistance through angiogenesis promotion, and accelerated tumor proliferation and metastasis (118).
2. BRD4 inhibition strategy: Wang et al. identified BRD4 as a critical regulator of macrophage senescence, pharmacological inhibition (JQ-1 or molibresib/I-BET762) or siRNA knockdown of BRD4 downregulates SASP factor expression, alleviates pathological lipid accumulation in aged macrophages, and reverses pro-tumorigenic functions (118).
3. Quercetin as a SASP modulator: Geng et al. demonstrated that quercetin significantly suppresses SASP components, including proinflammatory cytokines (IL-1α, IL-6, TNF-α, TGF-β), and matrix metalloproteinases (MMP2, MMP9, MMP12) inhibiting macrophage senescence (65), highlighting its potential as a novel SASP-targeted anti-tumor agent.

## 7 Conclusion

The relationship between senescent immune cells and tumor development involves multifaceted bidirectional interactions. Current research reveals that immunosenescent cells may promote tumor initiation and progression through multiple pathways: (1) impaired immune surveillance: functional decline in senescent immune cells diminishes tumor cell clearance capacity, enabling immune escape; (2) TME-mediated acceleration: tumor microenvironment (TME) factors accelerate immune cell senescence through cytokine/exosome secretion. This two-way effect not only affects the biological characteristics of tumor, but also provides a new perspective for us to understand the mechanism of tumor immune escape.

Immunosenescence demonstrates dualistic functions in oncology: (1) anti-tumor potential: proinflammatory SASP components may enhance antitumor immunity in specific contexts, (2) T cell suppression promotes tumor growth in other scenarios. Therefore, understanding this complex context-dependent roles is crucial to developing personalized cancer treatment plans.

As discussed above, targeting immunosenescence offers promising avenues, such as rejuvenation strategies: restoring immune cell function enhances antitumor responses and treatment efficacy; precision senotherapy: selective elimination of senescent immune cells within TME opens new frontiers for cancer immunotherapy. These approaches may improve both survival outcomes and quality of life (QoL) while reducing treatment-related toxicity.
